# Supplementary material for: An Immunohistochemical Study of MAGE Proteins in Hepatocellular Carcinoma
Source: Diagnostics (Basel). 2024 Aug 5;14(15):1692. doi: 10.3390/diagnostics14151692 (PMC11311968; doi:10.3390/diagnostics14151692)
Supplement: Supplementary file 1 [file diagnostics-14-01692-s001.zip › diagnostics-3041187-supplementary.pdf]

Table S1. Expression MAGE-C1 and MAGE-C2 (mean value ± standard deviation, SD) in relation to clinicohistopathological parameters of patients with hepatocellular carcinoma (p values were obtained using the Mann-Whithey U test).

	Total sample	MAGE-C1	P Value	MAGE-C2	P Value
Sex			**0.001**		**<0.001**
Female	17 (29.8)	2.53 ± 0.80		1.35 ± 0.78	
Male	40 (70.2)	1.68 ± 1.02		0.43 ± 0.74	
Age			0.135		0.104
<70 years	23 (40.4)	1.52 ± 1.38		0.96 ± 0.98	
≥70 years	34 (59.6)	2.21 ± 0.59		0.53 ± 0.75	
HBV or HCV			**0.008**		**<0.001**
Negative	45 (78.9)	1.78 ± 1.00		0.44 ± 0.69	
Positive	12 (21.1)	2.50 ± 1.00		1.67 ± 0.78	
Grade			**0.001**		**<0.001**
G1/G2	34 (59.6)	1.59 ± 1.05		0.35 ± 0.73	
G3	23 (40.4)	2.43 ± 0.79		1.22 ± 0.80	
Lymph Node Invasion			**0.001**		**<0.001**
No	18 (31.6)	1.22 ± 1.11		0.11 ± 3.23	
Yes	39 (68.4)	2.26 ± 0.82		0.97 ± 0.90	
Nodules			**<0.001**		**<0.001**
1	26 (45.6)	1.46 ± 0.95		0.15 ± 4.64	
2	31 (54.5)	2.32 ± 0.95		1.16 ± 0.86	
AFP			**<0.001**		**<0.001**
Low	25 (43.9)	1.28 ± 1.02		0.08 ± 0.28	
High	32 (56.1)	2.44 ± 0.72		1.19 ± 0.86	
Cirrhosis			0.064		**0.002**
No	43 (75.4)	1.79 ± 1.06		0.51 ± 0.83	
Yes	14 (24.6)	2.36 ± 0.84		1.29 ± 0.73	
Surgery			0.039		0.094
No	12 (21.1)	2.50 ± 0.52		1.00 ± 0.74	

Yes	45 (78.9)	1.78 ± 1.09		0.62 ± 0.89	
Status			**0.008**		**0.008**
Alive	16 (28.1)	1.31 ± 1.14		0.25 ± 0.68	
Dead	41 (71.9)	2.17 ± 0.89		0.88 ± 0.87	

Table S2. Positive (≥30%) expression of MAGE-C1 and MAGE-C2 in relation to clinicohistopathological parameters of patients with hepatocellular carcinoma (p values were obtained using the chi-square test).

		MAGE-C1				MAGE-C2	
	Positive (+)	OR (95% CI)	P Value		Positive (+)	OR (95% CI)	P Value
Sex			0.429				**<0.001**
Female	14 (82.4)	Ref.			14 (82.4)	Ref.	
Male	29 (72.5)	0.56 (0.136–2.35)			11 (27.5)	0.08 (0.02–0.34)	
Age			**0.001**				0,298
<70 years	12 (52.2)	Ref.			12 (52.2)	Ref.	
≥70 years	31 (91.2)	9.47 (2.24–39.98)			13 (38.2)	0.57 (0.19–1.66)	
HBV or HCV			**0.026**				**<0.001**
Negative	31 (68.9)	-			14 (31.1)	Ref.	
Positive	12 (100.0)	-			11 (91.7)	24.35 (2.86 – 207.48)	
Grade			**0.022**				**<0.001**
G1/G2	22 (64.7)	Ref.			7 (20.6)	Ref.	
G3	21(91.3)	5.72 (1.14–28.70)			18 (78.3)	13.89 (3.81–50.61)	
Lymph Node Invasion			**0.002**				**0.001**
No	9 (50.0)	Ref.			2 (11.1)	Ref.	
Yes	34 (87.2)	6.80 (1.82-25.38)			23 (59.0)	11.50 (2.31-57.10)	
Nodules			**0.026**				**<0.001**
1	16 (61.5)	Ref.			3 (11.5)	Ref.	
2	27 (87.1)	4.21 (1.13-15.70)			22 (71.0)	18.74 (4.48-78.42)	
AFP			**<0.001**				**<0.001**
Low	13 (52.0)	Ref.			2 (8.0)	Ref.	
High	30 (93.8)	13.84 (2.70-70.83)			23 (71.9)	29.38 (5.71-151.13)	
Cirrhosis			0.081				**<0.001**
No	30 (69.8)	Ref.			13 (30.2)	Ref.	
Yes	13 (92.9)	5.63 (0.66-47.67)			12 (85.7)	13.84 (2.70-70.83)	
Surgery			**0.026**				**0.014**
No	12 (100.0)	-			9 (75.0)	Ref.	
Yes	31 (68.9)	-			16 (35.6)	0.18 (0.04-0.78)	
Status			**0.036**				**0.003**
Alive	9 (56.3)	Ref.			2 (12.5)	Ref.	
Dead	34 (82.9)	3.78 (1.05-13.58)			23 (56.1)	8.94 (1.80 – 44.52)	

Table S3. Overall survival (OS) of patients with hepatocellular carcinoma in relation to the expression of MAGE-C1 and MAGE-C2.

	MAGE-C1 expression			MAGE-C2 expression			MAGE-C1 and MAGE-C2 co-expression		
	Negative (-)	Positive (+)		Negative (-)	Positive (+)		None (+)	One (+)	Both (+)
Number of patients	14	43		32	25		14	18	25
Survival rates									
6-month survival (%)	92.86±6.88	45.07±7.76		74.04±7.92	36.00±9.60		92.86±6.88	58.33±12.19	36.00±9.60
1-year survival (%)	54.17±13.81	10.91±6.48		36.90±9.76	6.75±5.96		54.17±13.81	16.20±13.30	6.75±5.96
Survival Time (months)									
Mean ± SE	12.42±1.04	6.56±0.72		10.31±0.87	5.38±0.81		12.42±1.04	7.55±0.76	5.38±0.81
95% CI	10.38-14.46	5.16-7.96		8.60-12.02	3.79-6.96		10.38-14.46	6.06-9.05	3.79-6.96
Median (95% CI)	14	5 (3.5-6.5)		10 (8-12)	5 (3.6-6.3)		14	8 (4.6-11.4)	5 (3.6-6.3)
Mortality (%)	7 (50.0%)	34 (79.1%)		18 (56.2%)	23 (92.0%)		7 (50.0%)	11 (61.1%)	23 (92.0%)
Hazard ratio (95% CI)									
Crude	4.05 (1.72-9.55)			3.23 (1,70-6,10)			Ref.	2.65 (1.00-7.08)	5.40 (2.23-13.08)
Adjusted	7.44 (1.96-28.27)			22.90 (6.67-78.60)			Ref.	1.66 (0.35-7.79)	29.82 (6.65-133.80)
P value (Log Rank test)	0.0003			0.0001				0.015 vs None (+)	<0.001 vs None (+)
									0.037 vs One (+)

Hazard ratio

p=0.001, p=0.003

p<0.001, p<0.001

p=0.053/p<0.001, p=0.519/p<0
